# Optimizing Sampling Design to Deal with Mist-Net Avoidance in Amazonian Birds and Bats

**DOI:** 10.1371/journal.pone.0074505

**Published:** 2013-09-18

**Authors:** João Tiago Marques, Maria J. Ramos Pereira, Tiago A. Marques, Carlos David Santos, Joana Santana, Pedro Beja, Jorge M. Palmeirim

**Affiliations:** 1 Departamento de Biologia Animal, Centro de Biologia Ambiental, Faculdade de Ciências, Universidade de Lisboa, Lisboa, Portugal; 2 Instituto de Desenvolvimento Sustentável Mamirauá, Tefé, Amazonas, Brazil; 3 Departamento de Biologia e Centro de Estudos do Ambiente e do Mar, Universidade de Aveiro, Campus Universitário de Santiago, Aveiro, Portugal; 4 Centre for Research into Ecological and Environmental Modelling, University of St Andrews, The Observatory, Buchanan Gardens, Fife, United Kingdom; 5 Department of Migration and Immuno-ecology, Max Planck Institute for Ornithology, Radolfzell, Germany; 6 ERENA, SA, Lisboa, Portugal; 7 CIBIO, Centro de Investigação em Biodiversidade e Recursos Genéticos, Universidade do Porto, Campus Agrário de Vairão, Vairão, Portugal; University of Regina, Canada

## Abstract

Mist netting is a widely used technique to sample bird and bat assemblages. However, captures often decline with time because animals learn and avoid the locations of nets. This avoidance or net shyness can substantially decrease sampling efficiency. We quantified the day-to-day decline in captures of Amazonian birds and bats with mist nets set at the same location for four consecutive days. We also evaluated how net avoidance influences the efficiency of surveys under different logistic scenarios using re-sampling techniques. Net avoidance caused substantial declines in bird and bat captures, although more accentuated in the latter. Most of the decline occurred between the first and second days of netting: 28% in birds and 47% in bats. Captures of commoner species were more affected. The numbers of species detected also declined. Moving nets daily to minimize the avoidance effect increased captures by 30% in birds and 70% in bats. However, moving the location of nets may cause a reduction in netting time and captures. When moving the nets caused the loss of one netting day it was no longer advantageous to move the nets frequently. In bird surveys that could even decrease the number of individuals captured and species detected. Net avoidance can greatly affect sampling efficiency but adjustments in survey design can minimize this. Whenever nets can be moved without losing netting time and the objective is to capture many individuals, they should be moved daily. If the main objective is to survey species present then nets should still be moved for bats, but not for birds. However, if relocating nets causes a significant loss of netting time, moving them to reduce effects of shyness will not improve sampling efficiency in either group. Overall, our findings can improve the design of mist netting sampling strategies in other tropical areas.

## Introduction

Birds and bats make up a great proportion of the vertebrate diversity in most terrestrial biomes. Both groups are particularly diverse in Neotropical rainforests [1], [2], so their study is essential to understand the functioning of these complex ecosystems. Many autecological and community studies in both groups require the capture of individuals, and mist netting has been extensively used for this purpose (e.g. [3]–[6]).

In the case of birds, species surveys are partly dependent on mist netting (e.g. [5], [7]–[9]), because in low visibility environments it complements visual and auditory methods. Netting efficiently detects secretive species and is not affected by inadequate knowledge of local bird calls or observer bias [10]. In addition, it has been demonstrated that, when used correctly, mist netting is a safe method to capture birds [11]. Bat studies are even more dependent on the use of mist netting, and almost all sampling of Neotropical forest bat assemblages has used this technique [6], [12]. Surveys using recordings of bat echolocation calls are becoming increasingly sophisticated [13], [14] and a few have been done in the Neotropics [15]–[17]. However, the results of these surveys depend greatly on the techniques and technology used [18], and the identification of the species emitting the calls is often difficult because of poor knowledge about the echolocation calls of most species and overlap in call structure [19]. In addition, Neotropical bat assemblages are dominated by Phyllostomids, which have calls that are difficult to detect in the field [20], [21].

The advantages of mist netting and the shortcomings of alternative methods warrant that netting will remain an essential technique in ecological studies of Neotropical birds and bats. However, one of its major drawbacks is that both birds and bats appear to learn the location of nets and thus avoid them, a phenomenon usually referred to as net avoidance or net shyness (e.g. [22]–[24]). It has been demonstrated that when nets are placed in the same location for consecutive days, net avoidance usually results in a substantial decline in captures over time [7], [25], leading to a decrease in the efficiency of sampling. The reduction in the numbers of captures can affect not only data collection about individual species or groups of species but also in community surveys, because the drop in captures usually results in the detection of fewer species [25].

Changing the locations of mist nets every day has been recommended as a strategy to avoid the decay in captures in both bats [26] and birds [8]. However, in some situations moving the nets may result in a loss of netting time, because the amount of work setting up mist nets at new sites can be substantial. This is the case if new suitable sites have to be selected, and net lanes have to be cleared for a large number of nets, or when using canopy nets, as their deployment is very time consuming [26]. For this reason, researchers need to weigh the advantages of moving the nets to avoid shyness against the consequences of loosing netting time.

In Neotropical studies the number of consecutive days with nets in the same locations is highly variable, both in birds and bats [6], [8]. A few of those studies quantify the day to day decay in the number of captures [25], [27], but they do not quantify the consequences of avoidance on the numbers of species detected. In addition, to our knowledge there are no data studies that evaluate the consequences of net avoidance on the efficiency of surveys.

The overall objectives of this paper are to: (i) quantify and analyse the effect of mist net avoidance on captures of Neotropical birds and bats, (ii) determine how net avoidance influences the efficiency of bird and bat surveys under different logistic scenarios, and (iii) formulate advice for designing sampling strategies that minimize the impact of net shyness on bird and bat sampling.

## Materials and Methods

### Study Area

Field work took place in the Amanã Sustainable Development Reserve (2°37′S, 64°37′W, Amazonas, Brazil), between April and December 2007. The Reserve includes over 2 million ha of forest including some that are seasonally flooded with nutrient-rich “white” water, known as várzea forests, and nutrient-poor “black” water, known as igapó forests [28], [29]. Canopy height varies among the three forest types but is usually between 15 and 35 m, with emergent trees often reaching 50 m [29]. The area receives about 2500 mm of annual precipitation, mostly during the high-water season, from January to June. The low-water season is usually between July and December. Water levels in flooded forests vary up to 10 meters between the two seasons [29].

### Bird and Bat Mist-netting

We captured birds and bats at a total of ten sites; four in non-flooded forest, three in igapó and three in várzea (for details, see [3], [4]). Each site was sampled in both the high-water and low-water seasons, resulting in a total of 20 sequences of four consecutive mist netting days. We assumed that birds and bats forget the location of the nets between the two seasons, because the time between visits averaged 173 days (range: 128–231), much longer than the three week interval recommended by Bierregaard [7]. In the high-water season nets were set just above the water in both várzea and igapó.

In each sampling site and occasion we used 10 mist nets (12×3 m; 5 shelves, Denier 110, 16 mm mesh size) for capturing both birds and bats in the forest understory. The same nets were opened for four consecutive days at the same locations between 6∶30 to 11∶00 and 16∶30 to 18∶30 (for birds), and between 18∶00 to 24∶00 (for bats), except when raining. Nets were checked every 30 min for birds and every 20 min for bats. All captured birds were identified, aged, sexed and marked by clipping the tip of the third primary of the right or left wing in the high- and low-water seasons, respectively. Bats were sexed, weighed and identified using the key by Lim & Engstrom [30] and an unpublished key by Erica Sampaio and Elisabeth Kalko. We marked the wing membranes of bats using a pen to recognize recaptures during the same four-day sequence. Our protocol was approved by the Brazilian CNPq and Ministério do Meio Ambiente.

### Testing the Effect of Capture Decay

We examined the trend of captures over four consecutive days using Generalized Estimating Equations (GEE; [31]) because of their suitability to analyse temporally correlated data. Rather than choose a specific correlation structure for the few (four) consecutive days sampled at each site, we used robust (and empirical) sandwich estimates of variance based on the correlation observed within sites to determine the standard errors for model parameters and any associated tests of significance, using the R package geepack [32].

### Influence of Species Abundance on Net Avoidance

We tested the relationship between species abundance and capture decay for all species with more than seven captures using a two-step approach. First, for each species, we determined the linear trend in captures over the four netting days using the pooled data of the 20 sampling sequences. Capture numbers where standardised (centred and divided by the standard deviation) to compare species with very different numbers of captures. Species with greater decay in captures have steeper trend slopes. We then tested the linear relationship between all these species-specific slope values and the logarithm of the numbers of animals captured. The significance of the relationship was determined by Ordinary Least Squares using PAST software - version 2.17b [33]. We assumed that the number of captures of each species is proportional to their local abundance in the forest understory, although this relationship is only approximate because it is affected by several confounding factors [34].

### Impact of Mist Net Avoidance on Sampling Efficiency

Mist net avoidance may affect sampling efficiency both by decreasing numbers of animals captured and the number of species detected. The decay in captures due to avoidance is likely to increase with the number of consecutive days that nets remain at the same locations. We quantified the effect of this decay for sampling strategies with nets remaining one, two, three and four consecutive days at the same locations, by restricting the capture data to the number of netting days that we would use in each strategy. For the one-day strategy (1-day), *i.e.* in the absence of net avoidance, we used the results of the first day of captures of the 20 sequences. For the three remaining strategies, which are presumably affected by increasing net avoidance effect, we used the first and second days of captures (2-day); the first, second and third days of captures (3-day); and all four days of captures (4-day). Using these values we then simulated a field season with 24 days and compared the results of the four strategies. We also analysed two scenarios: (i) when moving the nets to another sampling site does not imply the loss of netting time, and (ii) when moving the nets requires one field working day, as is often the case in logistically difficult study areas or when using canopy mist-nets.

To evaluate sampling success in terms of species captured we compared the efficiency of the four survey strategies –1-day, 2-day, 3-day and 4-day – with sample based species rarefaction curves. Calculations were done using the Mao Tao estimator on EstimateS (v. 8.2.0, [35]). The rarefaction curves were extrapolated to a total of 24 survey days using an estimator based on the Bernoulli product model, proposed by Colwell *et al.* [36]. The number of species present in the assemblage but not observed in any of the sampling units of the reference sample was obtained with the Chao2 estimator [37], [38]. All calculations were done separately for the three types of forest (non-flooded, várzea and igapó) and the results averaged.

## Results

### Quantification of the Decay in Captures

**Figure 1:**
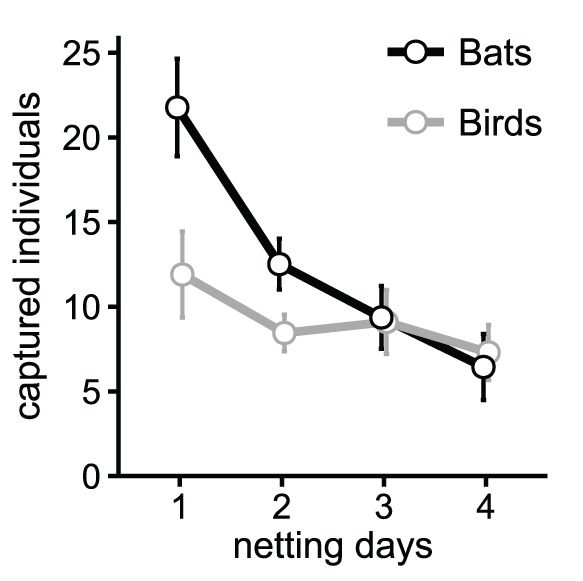
Decay in mist-net capture numbers of birds and bats. Mean daily capture numbers of bats and birds over four consecutive days with mist nets at the same location. Lines connect the average values over consecutive days. Data were pooled across seasons and forest types. Vertical lines represent 95% CI (*n* = 20).

Decay in captures with nets at the same locations was observed in both birds and bats, although it was greater in bats (Fig. 1). Captures over the 4-day period declined by 68% in bats and 45% in birds. Both declines were statistically significant (bats p<0.001; birds p = 0.013) and occurred mostly between the first and the second days of mist netting.

### Relationship between Species Abundance and the Decay in Captures

**Figure 2:**
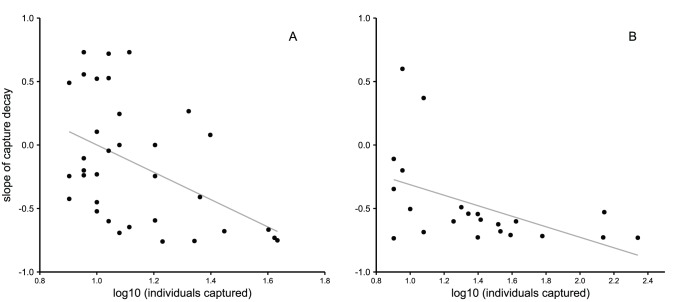
Relationship between species abundance and the decay in captures of birds (A) and bats (B). Data are the slope of the decay of captured individuals for each bird (A) and bat species (B) over 4 nights. The most abundant species tended to have a more accentuated decay.

The decay in captures was most evident in the common species of birds (Fig. 2A) and bats (Fig. 2B). This relationship was statistically significant for both groups (bats r = −0.49, p = 0.02; birds r = −0.46, p = 0.005).

### Influence of Net Avoidance on Bird and Bat Sampling

**Figure 3:**
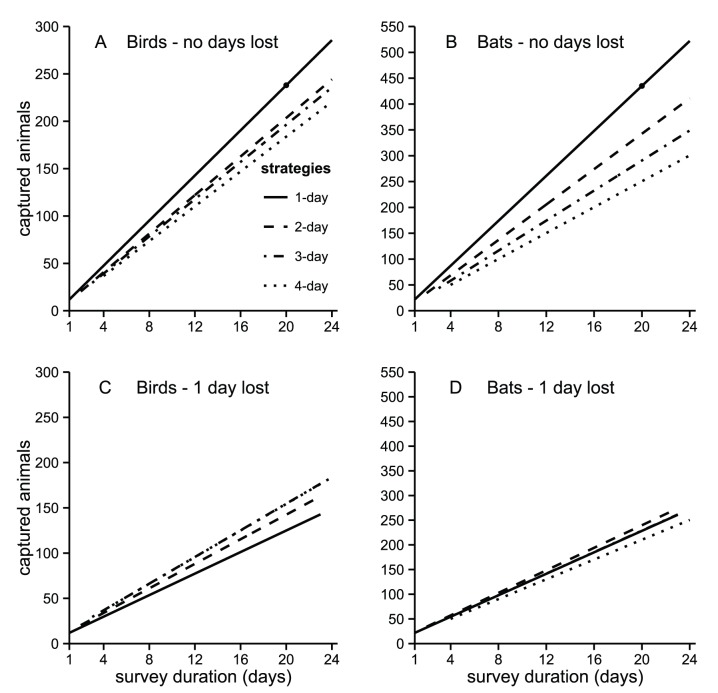
Capture numbers of birds (A,C) and bats (B,D) using different sampling strategies. Mist-net captures of birds and bats in simulated surveys lasting up to 24 days when nets were moved daily or remained at the same location 2, 3 or 4 days. When nets are set up in the same locations for 1 to 4 consecutive days net avoidance causes an increasing decline in the total number of animals captured in the survey of birds (A) and especially in bats (B). But whenever moving the nets involves losing one netting day per site, net avoidance does not affect the total numbers of animals captured in the survey of birds (C) and bats (D). The line representing the 1-day strategy was extrapolated to the right of the dot.

The comparison of the four sampling strategies (1-day, 2-day, 3-day and 4-day) shows that net avoidance had a strong impact on the efficiency of bird and bat surveys in terms of total number of captured individuals (Fig. 3). For the same overall survey duration we captured fewer animals if nets were deployed for more days at the same location. The drop in efficiency was greater for bats than for birds (Fig. 3A,B). For example, by changing the location of the nets daily at the end of 24 mist netting days we estimate we would have captured about 286 birds, whereas having the nets in the same location during four days would result in 221 captures, i.e. a 23% loss in efficiency. The same comparison in bats would result in an estimate of 522 versus 301 captures, a 42% drop in efficiency. However, this drop in efficiency only occurs if the locations of the nets can be changed without missing any mist netting days.

If moving the nets requires even just one field working day, as is often the case in logistically difficult study areas or when using canopy nets, then moving the nets daily may no longer be an advantage. For birds, the decrease in efficiency due to the loss of netting days is greater than the loss due to net avoidance (Fig. 3C). We estimate that keeping the nets at each location 3 days one would capture 184 birds in 24 field days, but only 149 if we move the nets daily with the loss of one field day in between. In bats the loss of capture days moving the nets cancels out the advantage of minimizing net avoidance and the results of all four sampling strategies become similar (Fig. 3D).

**Figure 4:**
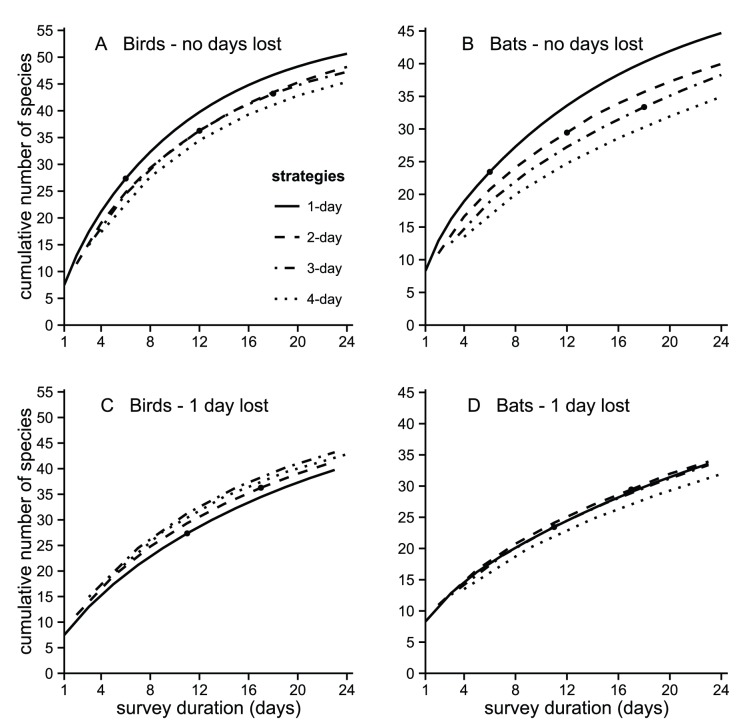
Species rarefaction curves for birds (A,C) and bats (B,D) obtained using four sampling strategies. Number of species detected in simulated surveys lasting up to 24 days when nets were moved daily or remained at the same location 2, 3 or 4 days. When nets are set up in the same locations on consecutive days, net avoidance causes an increasing decline in the number of species detected in the bird survey (A) and especially in bats (B). However, when moving the nets involves losing one field working day per site net avoidance does not affect the total numbers of species detected in the survey of birds (C) and bats (D). Above the black dots curves are extrapolated.

In the case of number of species detected, net avoidance had a substantial impact on the numbers for both birds and bats. Species rarefaction curves show that longer stays at each location result in less species recorded during a 24 day mist netting period (Fig. 4A,B). We estimate that without loss of days between netting locations only about 45 bird species are detected when the nets remain for 4-days at the same locations whilst moving the nets every day would result in the detection of 51 species, a difference of 12%. Likewise in bats the number of species detected would go up from 35 to 45, i.e. a difference of 22%. Again, the advantages of changing net locations daily disappear when moving them requires one field working day per site, because this reduces the time that is possible to dedicate to netting during the 24 day sampling period. In both birds and bats the numbers of species detected are more similar in all sampling strategies (Fig. 4C,D).

## Discussion

### Quantification of the Decay in Captures

The numbers of bats captured with the nets set up at the same locations over consecutive days dropped quickly from day to day. Captures in the second day were almost half of those in the first day, and by the fourth day they were reduced to less than one third. This pattern of decay is in line with that reported in other Neotropical and Temperate studies. Estrada et al. [39] and Simmons & Voss [25] reported an average decay of 50–70% between the first and the second day of netting in Mexico and French Guyana, respectively, while Esbérard [40] in South-Eastern Brazil observed a reduction of 65% between the first and third days. A similarly marked decay is evident in the few studies reporting quantitative observations in other biogeographic regions; for example, in Missouri (U.S.A.), Robbins et al. [41] observed a 45% decay between the first and second netting day. It can thus be concluded that net avoidance roughly halves the captures between the first and second day of captures, and reduces them further if nets remain for longer periods at the same location.

Mist net avoidance in Neotropical birds seems to be less accentuated than in bats. We observed an average drop of about 30% between the first and the second day using the same mist net setup. Data suggested a further decline with time, but less pronounced. Few studies report quantitative information on net avoidance in the Neotropics. However, Faaborg et al. [27] in Puerto Rico observed a 36% decline in captures between the first and second day, and of 14% from the second to the third day.

### Why is Net Avoidance so Marked in Bats?

Although the captures of both birds and bats declined over consecutive netting days, this decline was much steeper in the latter. In addition, far more birds than bats were recaptured in the same four-day netting sequence (13% vs 0.4%), adding to the evidence that bats are better than birds at learning to avoid previously encountered nets. Which factors may explain such strong net avoidance?

Bats are known to have an exceptionally good spatial memory [42]–[44], so once they have located a net they can probably avoid it easily. Larsen et al. [45] reported lower bat activity next to mist nets on the second and third nights of sampling. The detection of nets by microchiroptera is facilitated by the use of echolocation, which they use constantly or at least while flying in unfamiliar areas [46], but other factors may also help them in this process. It has been demonstrated that bats have a good capacity for social learning, taking clues from the observation of activities of other animals [47]. Because large numbers of bats tend to use the same commuting flyways [48], it is likely that individuals become aware of the presence of a mist-net by the observation of evasive flights of other bats. They may also locate nets when captured individuals are emitting distress calls, which are often loud and conspicuous [25], [49], [50].

The spatial ecology of most Amazonian bats may also help explain why they quickly learn the locations of mist nets. The great majority of the bats caught in mist nets in Neotropical rainforests are frugivorous and nectarivorous, and are known to use a trapline foraging strategy, i.e. they search for food along regularly used routes inspecting the same potential food sources in a sequential order [49], [51]. This repeated use of the same flying routes presumably helps them learn the location of mist nets.

### Relationship between Species Abundance and the Decay in Captures

There is little knowledge about the factors that make some species more prone than others to net avoidance. Our results show that in both birds and bats the most abundant species tend to show a steeper decline in the number of captures over time. Faaborg et al. [27] reported a similar pattern for birds in Puerto Rico. This is probably related to differences in the way various species use space and how it influences the probability of an individual being captured. Assuming that individuals have some capacity to learn the location of the nets [52], net avoidance should be more accentuated in species whose individuals have a greater chance of encountering a net. The risk of an individual bird being captured is thus potentially greater in species with small home ranges, in which the individual may quickly encounter a net placed within its limits [34]. Captures of these species are thus likely to drop rapidly over consecutive days of netting. Rarer species tend to have larger home ranges [53], [54], and are thus less likely to encounter a mist net set up in their range within the first day (or days). As a consequence the decay in captures in these species is potentially slower. However, although the correlation between capture decay and the number of captured individuals is significant the relationship is quite noisy. This may be explained by species-specific behavioural aspects that are known to influence net avoidance in birds, such as the species ability to notice, learn and remember the positions of the nets [22], and are also likely to affect bats. In the case of ecosystems with a strong vertical stratification, such as Neotropical rainforests, the way birds and bat species use vertical space [55], [56] is also likely to influence their probability of being trapped in mist nets [34].

### How to Deal with Mist Net Avoidance?

Field work is often costly and very time consuming, so sampling optimization is important in studies requiring the capture of large number of vertebrates. Although other studies demonstrated that the number of individuals captured can be affected by shyness, this is the first study that quantifies the effect of shyness on the number of species captured. The latter is particularly relevant in studies of the structure of species assemblages. Our results also revealed that shyness does not affect captures of all species equally, and is more severe in the commonest species. The systematic analyses of the consequences of shyness allow us to formulate advice for designing sampling strategies that minimize its impact on bird and bat sampling.

Although shyness affects captures of both birds and bats, the best strategy to minimize its consequences may not always be the same for the two groups. In addition, this strategy depends on the difficulty to reposition the nets within the study area and on the sampling objectives: *e.g*., to capture many individuals of common species or to characterize the area’s species assemblage.

When the number of mist nets in use is not large and the habitat is relatively open, the location of the nets can be changed quickly enough to avoid any loss of netting time. This is often also possible when nets are set up in pre-existing trails, thus avoiding the need to clear vegetation. In these situations researchers wanting to maximize the number of animals captured should move the nets daily. This strategy would yield gains of about 30% in birds and 70% in bats, compared to keeping nets four days at the same location. Our results indicate that moving the nets daily is particularly important when the aim is to capture individuals of common species, because their captures tend to be more affected by net-shyness.

If the objective of sampling is not to capture many individuals, but to assess the species present in an area, then different approaches should probably be used for sampling birds and bats. For bats it is still best to move the nets daily, because far more species will be detected (Fig. 4B). For birds the gain of moving the nets daily is modest (Fig. 4A). So, the practice of keeping them several days at the same locations, common in Neotropical bird studies [8], is not optimal but does not substantially decrease the survey efficiency. This is explained by the fact that most of the drop in captures is concentrated among the commonest species.

The need to open trails in the forest for the setting up of the mist nets or the installation of canopy mist nets often requires a substantial amount of field work. In these cases moving the nets may result in a loss of netting time and consequently in a reduction of captures. Our results show that if moving the nets causes the loss of one netting day per site then the resulting drop in bird captures is greater than that due to net shyness. Thus, sampling is more efficient if nets are kept at the same sites at least up to three days (Fig. 4C). In the case of bats the loss of captures due to the reduction in netting time is roughly equivalent to that due to net shyness, so frequent repositioning of nets does not reduce the sampling efficiency, although it also does not improve it.

This study is based on Neotropical data but its conclusions should help the design of sampling strategies elsewhere. This is because the overall drop in captures reported in temperate zones and in other tropical regions is not substantially different from what we observed [22], [41], [57]. However, researchers should take into consideration that net avoidance varies substantially among species and with environmental factors, and may even be negligible, as in the case of species on migration [22].

Net-shyness is not the only factor to be taken into consideration when deciding how frequently mist nets should be moved. For example, it may be important to maximize the number of independent replicates [58], and using nets at the same location for several days does not allow to treat each day as an independent replicate. Researchers may also want to ponder the negative impact of the clearing of vegetation to set up nets at a greater number of locations, which in some sites may not be negligible. However, it is evident that net shyness affects greatly the efficiency of sampling birds and bats in Neotropical forests, and our results should help researchers to design efficient sampling strategies, thus optimizing the use of limited research resources.
